# As a member of the All India Ophthalmological Society

**DOI:** 10.4103/0301-4738.67062

**Published:** 2010

**Authors:** K S Santhan Gopal

**Affiliations:** Kamalanethralaya, #81, First Floor, 4th Block, Koramangala, Bangalore - 560 034, India

Dear Editor,

As a member of the All India Ophthalmological Society (AIOS), a reviewer of the Indian Journal of Ophthalmology (IJO), and a member of the editorial board for a number of years, I admire and appreciate your tireless and efficient work. Your efforts have remarkably elevated the standard of the Journal.

I was interested to read the editorial, ″On being editor of (Indian) medical journal: A steeple chase″[1] wherein, you have unambiguously and thoughtfully laid out a path for the future editors to follow, and have clearly spelt out the hurdles a future editor is likely to encounter. You have spelt out the unnecessary and unavoidable tasks the editor of our journal has to contend with, besides the editor’s job. There are many historical and cultural reasons for that. It would be imprudent to go into the details of the reasons.

The quality of a journal is decided mostly by the quality of the articles appearing in the journal. The design of the cover pages adds to the attractiveness of the journal and the advertising pages certainly add to the coffers of the journal. The quality of printing, the timing of the journal, the regular appearance of the journal in pristine condition, in the hands of the members, and so on, all add to the value of the journal, albeit to a small extent. It is the impression of many of the readers that you have achieved a right balance of these variables and that is what has made the quality of the IJO go up a few notches.

We cannot always blame the members of AIOS for sending the article to foreign journals as a priority and this again has a historical reason, which is beyond the purview of the IJO or the AIOS.

I can only empathize with you when you state that there is no formal training of research at the undergraduate or even postgraduate level. It is preposterous, but true. What can an editor of a journal do about the medical education policy? He can, as an opinion maker, create awareness and draw the attention of the policy makers.

In many of the medical colleges, the postgraduates being guided by the misguided guides may not be universally true and I have many reasons to disagree. Our guides during our postgraduate days were, Prof. LP Agarwal, Prof. Madan Mohan, Prof. N.N. Sood, Prof. Premprakash, and many other stalwarts who ignited our brains. Maybe a few guides in some medical colleges, in some states, are incapable of guiding. I agree to an extent with Dr. SS. Hayreh when he states that, ″what matters in India is whom you know than what you know!″[2] However, to tar all the guides with the same brush, is doing a great injustice to the yeoman and selfless service many of our teachers and guides have been rendering and have rendered. I am most certain that the guidance you received during your postgraduate days, has certainly contributed to your professional achievements.

It is difficult to categorically state that there is lack of knowledge about ethics in publication among all the Indian authors. Maybe a few, but not necessarily among all. I understand your consternation, but truth is not what is opined in the editorial.

Many authors have no clue about the Helsinki declaration. Perhaps true, but then the Helsinki declaration itself has undergone a number of revisions and is mainly subservient to the medical and industrial interests of the powerful and rich countries. I cannot see how a declaration with no legal tooth can stop a rich country carrying out human experiments in poorer countries, with the connivance of the local, but corrupt government/s. What matters is not the awareness about some non-enforceable declaration, but conscientious attempts at finding a cure for the problem. I would rather have an Indian author conscientiously refuse to use a medication on a patient and be completely unaware of the Helsinki declaration, than injudiciously use a medication on a patient and be completely aware of all the declarations!

The editor’s office has to shift as the editor’s change every 3 or 6 years. That is the way we have our journal functioning and to be fair to the members of the society, they have always been astute in electing the right candidate for the right post. So, hope they continue to do so in the future as well. The journal has done well in spite of the editor being elected. I think the credit should go to the members of the society for voting intelligently.

Generating the funds for the journal is the collective responsibility of the society and its members. It should not be just the editor’s migraine.

I fully agree with you in your last few words. ″Most editors do leave an impact on the journal!″ Cannot be truer. And most will certainly do so in future as well. IJO is not just ready to pose, but is already posing a stiff challenge to the foreign counterparts. Thanks to your efforts and those of Dr. GN Rao, Dr. TP Das, and all the other editors before.
